# The association between chronic disease and depression in middle-aged and elderly people: The moderating effect of health insurance and health service quality

**DOI:** 10.3389/fpubh.2023.935969

**Published:** 2023-01-24

**Authors:** Dongxu Li, Min Su, Xi Guo, Bin Liu, Tianjiao Zhang

**Affiliations:** ^1^School of Public Administration, Inner Mongolia University, Hohhot, China; ^2^Inner Mongolia University of Technology, Hohhot, China

**Keywords:** chronic disease, multiple chronic diseases, depression, health insurance, health service quality

## Abstract

**Background:**

Depression in chronic disease patients was an important public health problem. However, limited work has been done on how to alleviate the depression of chronic disease patients. This paper attempted to explore the alleviating effect of health insurance and health service quality.

**Methods:**

A total of 11,500 middle-aged and elderly people were drawn from four waves (2011, 2013, 2015, and 2018) of the China Health and Retirement Longitudinal Study (CHARLS). We analyzed the effect of chronic disease on depression in middle-aged and elderly people in China, and explored the mechanism of action from health insurance and health service quality.

**Results:**

After adjusting for sociodemographic factors, any chronic disease (coefficient 1.471, *p* < 0.01) and multiple chronic diseases (coefficient 1.733, *p* < 0.01) could significantly increase the depression score. Any chronic disease increased the depression score (165.3 and 147.4% in non-health insurance group and health insurance group, respectively), the multiple chronic diseases increased the depression score (190.6 and 173.5% in non-health insurance group and health insurance group, respectively). Any chronic disease increased the depression score (161.3 and 139.5% in lower health service quality group and higher health service quality group, respectively), the multiple chronic diseases increased the depression score (228.4 and 162.9% in lower health service quality group and higher health service quality group, respectively). And similar results were obtained after using depression status instead of depression score.

**Conclusions:**

Chronic disease and multiple chronic diseases were important determinants of depression in middle-aged and elderly people. Health insurance and health service quality were the key factors in relieving the depression of chronic disease patients. Several strategies were urgently needed: paying attention to the mental health of chronic disease patients, increasing the participation rate of health insurance, further improving the quality of health service, and alleviating the psychological harm caused by chronic disease.

## 1. Introduction

With the rapid economic development and changes in lifestyle, the spectrum of human diseases has changed, and the main threat to human health has gradually transformed from traditional infectious diseases to chronic non-communicable diseases. The “Report on the Nutrition and Chronic Disease Status of Chinese Residents (2020)” mentioned that the number of chronic disease patients in China would continue increasing, and the deaths caused by chronic disease accounted for 88.5% of the total deaths in 2019 (1). Due to the multiple pathogenic factors and a long course of chronic disease, patients may also suffer from multiple chronic diseases simultaneously, leading to a more serious health crisis (2). Treating chronic disorders was regarded as an important policy goal in the health system.

It was not only necessary to pay attention to the physical health of chronic disease, but also to the harm to the mental health. After suffering from chronic disease, patients faced both financial burden and psychological pressure. Depression was a mental illness that accompanied chronic disease patients, and as the number of chronic diseases increased, the risk of depression increased (3). And depression was highly associated with chronic disease, poor quality of life and suicide (4). Therefore, it was necessary to explore the association between chronic disease, multiple chronic diseases and depression, and to discover the corresponding mitigation mechanisms.

For patients with chronic disease and depression, the financial burden was an important issue they faced. Health insurance was a useful tool to help people deal with this financial stress (5). For medical institutions, high-quality health service was the factor that alleviating the suffering of patients. Medical coverage could lead to the expected health outcomes only if the quality of health service was guaranteed (6). And early intervention and effective health service and comprehensive care were necessary (7). This paper would discuss the alleviating effect of health insurance and health service quality on the association between chronic disease and depression, hoping to provide reference for reducing the harm of chronic disease and formulating more targeted prevention and control measures.

## 2. Literature review

The previous studies have shown a significant positive correlation between having chronic disease and the risk of depression (8–10). In terms of the role of health insurance, some scholars believed that it could alleviate the harm of diseases by sharing financial pressure. For example, Baicker et al. (11) pointed that health insurance increased the consumption of the commonly used drugs for the treatment of depression, met the needs of patients for mental health care, and significantly promoted the diagnosis and treatment of depression. Fry and Sommers (12) argued that health insurance improved access to treatment and medication for people with depression, even in area where there was a relative shortage of mental health professionals. Some scholars have explored the effect of different health insurance. Peng and Zhu (5) stated that social health insurance reduced the financial stress in outpatients, and private health insurance was found to be a positive predictor of financial stress in hospitalized chronic disease patients. Some scholars believed that health insurance could promote medical treatment. For example, Adu (13) found that chronic disease patients who participated in health insurance were more likely to attend a regular medical institutions for treatment, and were less likely to attend a self-care. Skinner et al. (14) pointed that the presence of health insurance generally increased the utilization of health service by people with chronic disease. Geng et al. (15) had further pointed that patients' cognition of health insurance policies, the expected realization of insurance plans, and patients' perceived value of insurance coverage would effectively improve patient satisfaction.

Regarding health service quality, some scholars believed that it was directly related to disease hazards. For example, Jafree (16) applied bivariate regression analysis and found that when health service was insufficient or dissatisfied, patients would face greater depression. Arabyat and Raisch (17) described that the health service quality of diagnosis and assessment by healthcare providers was an important part of the care of patients with chronic obstructive pulmonary disease, and contributed to improving functioning and overall health-related quality of life. Gong et al. (18) believed that patient satisfaction with the quality of health service was closely related to the availability and affordability of health service, waiting times, and the treatment environment. Sibbritt et al. (19) pointed that when recommending treatments for depression, healthcare practitioners needed to prioritize the availability of health service to ensure equity in healthcare. Howse (20) argued that health service helped drive improvements in chronic disease prevention and management and promoted healthy aging.

To sum up, scholars have conducted extensive research on the association between chronic disease and depression, and have also tried to explore the role of health insurance and health service. However, on the one hand, there were few studies on the mitigation measures of depression in chronic disease, especially the lack of empirical testing. On the other hand, chronic disease was major public health problem that concerned families and society, and required the support of health insurance and high-quality health service. Nevertheless, few studies have explored the association between chronic disease and depression in the public health system from these two aspects. Therefore, this paper tried to explore the mechanism of action from health insurance and health service quality on the basis of discovering the association between chronic disease and depression, and to provide empirical evidence for alleviating the depressive harm of chronic disease.

## 3. Data and methods

### 3.1. Data and participants

Data came from the 2011–2018 CHARLS, a nationally representative survey that aimed to collect a set of high-quality microdata representing households and individuals aged 45 and above in China. And the data aimed to analyze the problem of population aging in China and promote interdisciplinary research on the problem of aging. CHARLS also included an assessment of the social, economic, and health status of community residents (21). The national baseline survey for CHARLS was conducted in 2011, covering 150 county-level units, 450 village-level units, and about 17,000 people in 10,000 households. Since 2011, CHARLS has conducted four follow-up surveys. In this study, middle-aged and elderly people aged 45 years and above were selected as the research participants. And there was a sample of 11,500 people who participated in four surveys in 2011, 2013, 2015, and 2018 to conduct panel data analysis.

### 3.2. Variables

The dependent variable in this paper was depression score. Depression was assessed using the CES-D10. The CES-D-10 response scale included 10 questions about the participants' experiences of feeling bothered, having trouble in concentrating, etc. over the past week. Participants were asked to answer each question according to the Chinese version of the scale: 0 = 1, 1 = 1–2, 2 = 3–4, and 3 = 5–7 days. A summary score was generated by summing up the score of each question (the scale of the two positive items was reversed before summing up). Total CES-D-10 score ranged from 0 to 30, with higher score indicating more serious depressive symptoms.

Whether you had chronic disease and whether you had multiple chronic diseases were the independent variables, and were represented by the answer to “Have you been diagnosed with conditions listed below by a doctor?” CHARLS asked about 14 types of chronic diseases diagnosed by doctors, such as hypertension, dyslipidemia, diabetes or high blood sugar, cancer or malignant tumor (excluding minor skin cancers), chronic lung diseases, such as chronic bronchitis, emphysema (excluding tumors, or cancer), liver disease (except fatty liver, tumors, and cancer), heart attack, coronary heart disease, angina, congestive heart failure, or other heart problems, stroke, kidney disease (except for tumor or cancer), stomach or other digestive disease (except for tumor or cancer), emotional, nervous, or psychiatric problems, memory-related disease, arthritis or rheumatism and asthma. Chronic disease referred to patients with at least one chronic disease, and multiple chronic diseases referred to patients with at least two types of chronic diseases. The moderating variables were whether to participate in health insurance and the quality of health service. The health insurance data came from the question and answer of “Are you the policy holder/primary beneficiary of any of the types of health insurance listed below?” in the questionnaire. The data on the quality of health service came from the questionnaire “Are you satisfied with the quality, cost, and convenience of local health care services?” The measurement index of health service quality came from the subjective cognitive responses of patients, which was an effective expression of the health service received by social individuals. In this paper, the health service quality was set as a dichotomous variable, and those who answered “somewhat dissatisfied” and “very dissatisfied” were the group with lower service quality, and assigned a value of 0, and those who answered “very satisfied,” “somewhat satisfied,” and “neutral” were the group with higher quality, and assigned a value of 1. The 2011 and 2013 databases did not include the satisfaction of health service quality, so the data from 2015 and 2018 were used to measure the health service quality. In addition, the paper controlled for variables such as gender, age, education, marital status, income, place of residence, region, smoke, drink, social, sports, and sleep time.

### 3.3. Methods

Categorical variables were described as percentages, and continuous variables were described as mean and standard deviation. When the outcome variable was depression score, this study used the OLS model for regression analysis. When the outcome variable was depression status, this study used the Probit model for regression analysis. In addition, due to the self-selection effect of health insurance, this study used the Heckman model for analysis. Since health insurance and health service quality were both dichotomous categorical variables, according to the introduction of moderation regression by Wen et al. (22) and Yang et al. (23), this paper used the method of group regression to test the moderating effect. All data were statistically analyzed using STATA 15.1 software.

## 4. Results

### 4.1. Descriptive statistics

#### 4.1.1. Socio-demographic characteristics

This study included 11,500 respondents (Table 1), and rural samples (75.6%) accounted for a larger proportion. There were slightly more females (52.9%) than males, and the average age was 58.479. The sample had an educational level at primary school or below (67.9%) and most were married (89.3%). There were slightly more respondents in the eastern region (34.5%). In terms of lifestyle, the sample had a lower proportion of participant who smoking (39.0%), drinking (33.4%), doing sports (36.8%), and a higher proportion of participant had social behavior (52.6%). The average sleep time was 6.902 h. More details could be found in Table 1.

**Table 1:** Basic socio-demographic characteristics (2011, *N* = 11,500).

**Variable**	**Total**		**Rural**		**Urban**	
	***N***	**%**	***N***	**%**	***N***	**%**
**Basic characteristics**						
**Gender**						
Male	5,413	47.1	4,124	47.4	1,289	46.0
Female	6,080	52.9	4,568	52.5	1,512	54.0
Missing value	7	0.1	7	0.1	0	0
**Age**						
Mean ± S.D.	58.479 ± 8.937		58.613 ± 8.927		58.062 ± 8.958	
Missing value	22	0.2	17	0.1	5	0.2
**Education**						
Primary school and below	7,811	67.9	6,464	74.3	1,347	48.1
Junior high school	2,403	20.9	1,625	18.7	778	27.8
High school and above	1,275	11.1	604	6.9	671	24.0
Missing value	11	0.1	6	0.1	5	0.2
**Marital status**						
Married	10,275	89.3	7,780	89.4	2,495	89.1
Others	1,225	10.7	919	10.6	306	10.9
**Income (yuan)**						
Mean ± S.D.	10,663.955 ± 31,145.955		7,136.848 ± 18,417.755		21,611.366 ± 52,634.849	
Missing value	34	0.3	27	0.3	7	0.2
**Region**						
Eastern region	3,971	34.5	3,075	35.3	869	31.0
Central region	3,778	32.9	2,778	31.9	1,000	35.7
Western region	3,751	32.6	2,846	32.7	905	32.3
**Lifestyle**						
**Smoke**						
Yes	4,484	39.0	3,482	40.0	1,002	35.8
No	7,014	61.0	5,215	60.0	1,799	64.2
Missing value	2	0.1	2	0.1	0	0
**Drink**						
Yes	3,836	33.4	2,939	33.8	897	32.0
No	7,657	66.6	5,754	66.1	1,903	67.9
Missing value	7	0.1	6	0.1	1	0.1
**Social**						
Yes	6,044	52.6	4,330	37.7	1,714	61.2
No	5,456	47.4	4,369	62.3	1,087	38.8
**Sports**						
Yes	4,227	36.8	3,199	27.8	1,028	36.7
No	7,273	63.2	5,500	62.2	1,773	63.3
**Sleep time (hours)**						
Mean ± S.D.	6.902 ± 1.988		6.896 ± 2.037		6.923 ± 1.829	
Missing value	845	7.3	636	7.3	199	7.1
N	11,500	100	8,699	75.6	2,801	24.4

#### 4.1.2. Development trend of key variables

Total CES-D-10 score ranged from 0 to 30. The mean depression score of middle-aged and elderly people in 2011 was 8.378 (8.813 and 7.030 in rural and urban, respectively), and the mean depression score in middle-aged and elderly people in 2013 was 7.902 (8.254 and 6.840 in rural and urban, respectively), and the mean depression score in middle-aged and elderly people in 2015 was 8.170 (rural and urban were 8.617 and 6.819, respectively), and the mean depression score of middle-aged and elderly people in 2018 was 8.800 (rural and urban were 9.220 and 7.587, respectively).

Previous studies suggested that 10 was the optimal cutoff value for CES-D-10 (24, 25). We used this cutoff to generate the dichotomous depression status variable (0 normal, 1 depression), and the score of ≥10 was deemed to have depression. The prevalence rate of depression among the middle-aged and elderly in 2011 was 36.8% (39.5 and 28.4% in rural and urban, respectively), and in 2013, the prevalence was 31.7% (34.0 and 24.8% in rural and urban, respectively). The prevalence of depression in middle-aged and elderly people was 34.6% in 2015 (37.4 and 26.0% in rural and urban, respectively), and the prevalence of depression was 38.7% in 2018 (41.3 and 31.4% in rural and urban, respectively).

The prevalence of chronic disease among the middle-aged and elderly in 2011 was 67.0% (66.7 and 68.0% in rural and urban, respectively), and in 2013, the prevalence was 67.1% (66.8 and 68.0% in rural and urban, respectively). The prevalence of chronic disease in middle-aged and elderly people was 73.9% in 2015 (73.7 and 74.3% in rural and urban, respectively), and the prevalence was 76.7% in 2018 (76.4 and 77.6% in rural and urban, respectively).

The prevalence of multiple chronic diseases among the middle-aged and elderly in the 2011 sample was 36.8% (36.0 and 39.1% in rural and urban, respectively), and the prevalence was 37.1% (36.4% in rural and 39.3% in urban, respectively) in 2013. In 2015, the prevalence of multiple chronic diseases in middle-aged and elderly people was 45.1% (44.3% in rural 47.4% in urban), and the prevalence was 46.4% in 2018 (45.7% in rural and 48.5% in urban).

In 2011, the proportion of middle-aged and elderly people participating in health insurance was 94.6% (95.4% in rural and 92.0% in urban), and 96.9% in 2013 (97.0% in rural and 96.8% in urban). In 2015, the proportion was 92.1% (92.3 and 91.4% in rural and urban, respectively), and 97.2% in 2018 (96.9 and 98.1% in rural and urban).

The health service quality results were distributed between 0 and 1. The mean value of the health service quality was 0.811 in 2015 (0.818 and 0.787 in rural and urban, respectively), and the mean value of the health service quality was 0.838 in 2018 (0.848 for rural and 0.810 for urban). More details could be found in Table 2.

**Table 2:** Key variables and trends.

**Variables**		**2011**	**2013**	**2015**	**2018**
Depression score	Total	8.378	7.902	8.170	8.800
	Rural	8.813	8.254	8.617	9.220
	Urban	7.030	6.840	6.819	7.587
The prevalence rate of depression	Total	0.368	0.317	0.346	0.387
	Rural	0.395	0.340	0.374	0.413
	Urban	0.284	0.248	0.260	0.314
Chronic disease	Total	0.670	0.671	0.739	0.767
	Rural	0.667	0.669	0.737	0.764
	Urban	0.680	0.680	0.743	0.776
Multiple chronic diseases	Total	0.368	0.371	0.451	0.464
	Rural	0.360	0.364	0.443	0.457
	Urban	0.391	0.393	0.474	0.485
Health insurance	Total	0.946	0.969	0.921	0.972
	Rural	0.954	0.970	0.923	0.969
	Urban	0.920	0.968	0.914	0.981
Health service quality	Total			0.811	0.838
	Rural			0.818	0.848
	Urban			0.787	0.810

### 4.2. Influencing factors

#### 4.2.1. The influence of chronic disease on depression

Model 1 was the regression of the control variable on the depression score. The results showed that *P*-valve was < 0.001 and *R*^2^ was 0.151, the model was statistically significant and the model fit was good. Model 2 added chronic disease, and the results showed that compared with those without chronic disease, middle-aged and elderly people with chronic disease had a 147.1% increase in depression score. Model 3 was the regression of multiple chronic diseases and control variables on the depression score. The results showed that compared with those without multiple chronic diseases, middle-aged and elderly people with multiple chronic diseases had a 173.3% increase in depression score. Model 4 and Model 5 were regression results using the Heckman model, Model 4 was the regression of control variables on the health insurance. Model 5 was the regression of chronic disease and control variables on the depression status. The results showed that after adding Inverse Mills Ratio (IMR), middle-aged and older adults with chronic disease had higher depression prevalence, and having chronic disease was a major determinant of depression. More details could be found in Table 3.

**Table 3:** Analysis of the influence of chronic disease on depression.

**Variables**	**Model 1**	**Model 2**	**Model 3**	**Model 4**	**Model 5**
Chronic disease		1.471^***^		0.127^***^	0.440^***^
		(0.077)		(0.033)	(0.026)
Multiple chronic diseases			1.733^***^		
			(0.080)		
Health insurance (Ref: Not have)	−0.243^*^		−0.281^**^	−0.313^**^	−0.078^*^
	(0.142)		(0.142)	(0.142)	(0.042)
**Year (Ref: 2011)**					
2013	−0.405^***^	−0.386^***^	−0.384^***^	0.381^***^	−0.168^***^
	(0.062)	(0.062)	(0.062)	(0.039)	(0.032)
2015	−0.121^*^	−0.188^***^	−0.217^***^	−0.217^***^	−0.093^***^
	(0.068)	(0.068)	(0.068)	(0.033)	(0.031)
2018	0.539^***^	0.453^***^	0.464^***^	0.377^***^	0.101^***^
	(0.085)	(0.085)	(0.084)	(0.043)	(0.034)
Gender (Ref: Female)	−1.644^***^	−1.587^***^	−1.544^***^	0.081^*^	−0.405^***^
	(0.122)	(0.120)	(0.119)	(0.045)	(0.035)
Age	0.003	−0.005	−0.009^**^	−0.002	−0.003^**^
	(0.005)	(0.005)	(0.005)	(0.002)	(0.002)
**Education (Ref: Primary school and below)**					
Junior high school	−0.982^***^	−0.921^***^	−0.915^***^	0.239^***^	−0.204^***^
	(0.108)	(0.106)	(0.105)	(0.044)	(0.035)
High school and above	−1.411^***^	−1.350^***^	−1.401^***^	0.216^***^	−0.318^***^
	(0.133)	(0.131)	(0.129)	(0.058)	(0.045)
Marital status (Ref: Others)	−1.349^***^	−1.368^***^	−1.371^***^	0.348^***^	−0.291^***^
	(0.133)	(0.132)	(0.131)	(0.042)	(0.046)
Income	−0.110^***^	−0.110^***^	−0.110^***^	0.005	−0.029^***^
	(0.010)	(0.010)	(0.010)	(0.004)	(0.003)
Place of residence (Ref: Rural)	−1.092^***^	−1.124^***^	−1.154^***^	−0.190^***^	−0.304^***^
	(0.101)	(0.100)	(0.099)	(0.038)	(0.033)
**Region (Ref: Eastern region)**					
Central region	1.247^***^	1.139^***^	1.114^***^	0.093^**^	0.318^***^
	(0.102)	(0.100)	(0.100)	(0.038)	(0.030)
Western region	1.651^***^	1.506^***^	1.436^***^	0.024	0.414^***^
	(0.105)	(0.104)	(0.103)	(0.038)	(0.030)
Sleeping time	−0.544^***^	−0.536^***^	−0.534^***^	−0.010	−0.142^***^
	(0.017)	(0.017)	(0.017)	(0.007)	(0.005)
Sports (Ref: No)	−0.098^*^	−0.104^*^	−0.107^*^	0.089^***^	−0.022
	(0.060)	(0.060)	(0.060)	(0.029)	(0.021)
Smoke (Ref: No)	0.476^***^	0.447^***^	0.443^***^	−0.029	0.104^***^
	(0.117)	(0.115)	(0.114)	(0.043)	(0.034)
Drink (Ref: No)	−0.182^**^	−0.146^**^	−0.142^**^	0.041	−0.049^**^
	(0.073)	(0.073)	(0.072)	(0.035)	(0.024)
Social (Ref: No)	−0.442^***^	−0.457^***^	−0.459^***^	0.132^***^	−0.100^***^
	(0.056)	(0.056)	(0.056)	(0.028)	(0.021)
IMR					0.474
					(0.774)
*P*	< 0.001	< 0.001	< 0.001	< 0.001	< 0.001
*R^2^*	0.151	0.167	0.175	0.016	0.121
*N*	38667	38667	38667	42841	38667

Standard errors in parentheses ^*^p < 0.1, ^**^p < 0.05, ^***^p < 0.01.

#### 4.2.2. Adjustment regression of health insurance

Depression score was used as the dependent variable, chronic disease as the independent variable, and health insurance as the moderator variable to test the mechanism of action. Model 6 was the regression of chronic disease on depression score in the non-health insurance group. It could be seen that chronic disease in the non-health insurance group increased the depression score by 165.3%. Model 7 was the regression of chronic disease on depression score in the health insurance group. The results showed that chronic disease in the health insurance group increased the depression score by 147.4%, that was, participating in health insurance could reduce the depression score of chronic disease patients. Model 8 was the regression of multiple chronic diseases on depression score in the non-health health insurance group. The results showed that the multiple chronic diseases in the non-health insurance group increased the depression score by 190.6%. Model 9 was the regression of multiple chronic diseases on depression score in the health insurance group. The results showed that the chronic disease in the health insurance group increased the depression score by 173.5%, that was, participating in health insurance could reduce the depression score of patients with multiple chronic diseases. More details could be found in Table 4. The same steps of regression were performed using depression status instead of depression score, and it was found that there was the same moderating effect in the relationship between multiple chronic diseases and depression status (47.9 > 45.0%), while there was no such moderating effect in the relationship between chronic disease and depression status (40.8 < 44.2%). More details could be found in Table 5.

**Table 4:** Adjusted regression results of health insurance.

	**Depression score**			
	**Model 6**	**Model 7**	**Model 8**	**Model 9**
	**Non–health insurance**	**Health insurance**	**Non–health insurance**	**Health insurance**
Chronic disease	1.653^***^	1.474^***^		
	(0.342)	(0.078)		
Multiple chronic diseases			1.906^***^	1.735^***^
			(0.353)	(0.081)
**Year (Ref:2011)**				
2013	0.336	−0.414^***^	0.307	−0.412^***^
	(0.396)	(0.063)	(0.396)	(0.063)
2015	−0.237	−0.187^***^	−0.246	−0.217^***^
	(0.350)	(0.070)	(0.347)	(0.070)
2018	0.312	0.455^***^	0.343	0.464^***^
	(0.532)	(0.086)	(0.531)	(0.086)
Gender (Ref: Female)	−1.149^**^	−1.596^***^	−1.023^**^	−1.556^***^
	(0.469)	(0.121)	(0.469)	(0.120)
Age	−0.006	−0.005	−0.007	−0.009^*^
	(0.019)	(0.005)	(0.019)	(0.005)
**Education (Ref: Primary school and below)**				
Junior high school	−1.118^***^	−0.908^***^	−1.178^***^	−0.901^***^
	(0.405)	(0.107)	(0.402)	(0.106)
High school and above	−1.185^**^	−1.344^***^	−1.375^**^	−1.392^***^
	(0.558)	(0.131)	(0.549)	(0.130)
Marital status (Ref: Others)	−1.498^***^	−1.368^***^	−1.360^***^	−1.376^***^
	(0.450)	(0.135)	(0.448)	(0.135)
Income	−0.163^***^	−0.108^***^	−0.156^***^	−0.109^***^
	(0.037)	(0.010)	(0.037)	(0.010)
Place of residence (Ref: Rural)	−1.135^***^	−1.129^***^	−1.191^***^	−1.157^***^
	(0.375)	(0.101)	(0.375)	(0.100)
**Region (Ref: Eastern region)**				
Central region	0.686^*^	1.152^***^	0.683^*^	1.128^***^
	(0.401)	(0.101)	(0.398)	(0.100)
Western region	0.669^*^	1.535^***^	0.642^*^	1.463^***^
	(0.390)	(0.105)	(0.389)	(0.104)
Sleeping time	−0.603^***^	−0.541^***^	−0.591^***^	−0.539^***^
	(0.074)	(0.017)	(0.075)	(0.017)
Sports (Ref: No)	−0.096	−0.127^**^	−0.079	−0.130^**^
	(0.308)	(0.061)	(0.308)	(0.061)
Smoke (Ref: No)	0.266	0.449^***^	0.180	0.448^***^
	(0.449)	(0.116)	(0.448)	(0.115)
Drink (Ref: No)	−0.211	−0.157^**^	−0.208	−0.152^**^
	(0.376)	(0.074)	(0.374)	(0.073)
Social (Ref: No)	−0.898^***^	−0.451^***^	−0.865^***^	−0.454^***^
	(0.296)	(0.058)	(0.295)	(0.057)
*P*	< 0.001	< 0.001	< 0.001	< 0.001
*R^2^*	0.146	0.168	0.150	0.176
*N*	1,721	36,946	1,721	36,946

Standard errors in parentheses ^*^p < 0.1, ^**^p < 0.05, ^***^p < 0.01. The table showed the moderating effect of health insurance on the relationship between chronic diseases and depression score.

**Table 5:** Adjusted regression results of health insurance.

	**Depression status**			
	**Non–health insurance**	**Health insurance**	**Non–health insurance**	**Health insurance**
Chronic disease	0.408^***^	0.442^***^		
	(0.100)	(0.025)		
Multiple chronic diseases			0.479^***^	0.450^***^
			(0.100)	(0.023)
**Year (Ref: 2011)**				
2013	0.024	−0.191^***^	0.015	−0.191^***^
	(0.128)	(0.023)	(0.128)	(0.023)
2015	−0.020	−0.082^***^	−0.027	−0.090^***^
	(0.102)	(0.024)	(0.103)	(0.024)
2018	0.103	0.086^***^	0.110	0.090^***^
	(0.147)	(0.027)	(0.148)	(0.027)
Gender (Ref: Female)	−0.307^**^	−0.410^***^	−0.278^**^	−0.402^***^
	(0.128)	(0.036)	(0.128)	(0.035)
Age	−0.007	−0.003^*^	−0.008	−0.004^**^
	(0.006)	(0.002)	(0.006)	(0.002)
**Education (Ref: Primary school and below)**				
Junior high school	−0.244^*^	−0.210^***^	−0.262^**^	−0.208^***^
	(0.133)	(0.032)	(0.134)	(0.032)
High school and above	−0.345^**^	−0.325^***^	−0.396^**^	−0.338^***^
	(0.175)	(0.044)	(0.177)	(0.043)
Marital status (Ref: Others)	−0.314^***^	−0.308^***^	−0.284^**^	−0.309^***^
	(0.118)	(0.035)	(0.118)	(0.035)
Income	−0.034^***^	−0.030^***^	−0.032^***^	−0.030^***^
	(0.011)	(0.003)	(0.011)	(0.003)
Place of residence (Ref: Rural)	−0.345^***^	−0.296^***^	−0.362^***^	−0.303^***^
	(0.110)	(0.031)	(0.112)	(0.030)
**Region (Ref: Eastern region)**				
Central region	0.173	0.317^***^	0.173	0.314^***^
	(0.112)	(0.030)	(0.113)	(0.030)
Western region	0.220^**^	0.420^***^	0.215^*^	0.405^***^
	(0.110)	(0.030)	(0.110)	(0.030)
Sleeping time	−0.142^***^	−0.143^***^	−0.140^***^	−0.142^***^
	(0.021)	(0.005)	(0.021)	(0.005)
Sports (Ref: No)	−0.034	−0.031	−0.035	−0.031
	(0.091)	(0.021)	(0.091)	(0.021)
Smoke (Ref: No)	0.037	0.107^***^	0.011	0.107^***^
	(0.122)	(0.034)	(0.123)	(0.034)
Drink (Ref: No)	−0.004	−0.058^**^	−0.001	−0.057^**^
	(0.107)	(0.024)	(0.108)	(0.024)
Social (Ref: No)	−0.128	−0.106^***^	−0.120	−0.106^***^
	(0.087)	(0.019)	(0.087)	(0.019)
*P*	< 0.001	< 0.001	< 0.001	< 0.001
*R^2^*	0.105	0.122	0.109	0.126
*N*	1,721	36,946	1,721	36,946

Standard errors in parentheses ^*^p < 0.1, ^**^p < 0.05, ^***^p < 0.01. The table showed the moderating effect of health insurance on the relationship between chronic diseases and depression status.

#### 4.2.3. Adjustment regression of health service quality

Taking depression score as dependent variable, chronic disease as independent variable, and health service quality as a moderator variable to test the mechanism of action. Model 10 was the regression of chronic disease on depression score in the lower health service quality insurance group. The results showed that the chronic disease in the lower health service quality group increased the depression score by 161.3%. Model 11 was the regression of chronic disease on depression score in the higher health service quality group. The results showed that the chronic disease in the higher health service quality group increased the depression score by 139.5%. The improvement of the health service quality could reduce the depression score of the chronic disease patients. Model 12 was the regression of multiple chronic diseases on depression score in the lower health service quality group. The results showed that the multiple chronic diseases in the lower health service quality group increased the depression score by 228.4%. Model 13 was the regression of multiple chronic diseases on depression score in the higher health service quality group. The results showed that the multiple chronic diseases in the higher health service quality group increased the depression score by 162.9%, that was, improving the quality of health service could reduce depression score in patients with multiple chronic diseases. More details could be found in Table 6. Using depression status instead of depression score, and performed regression with the same steps, and obtained the same results (50.8 > 38.6%, 55.8 > 37.3%). That was, improving the quality of health service could reduce the prevalence of depression in patients with chronic disease or multiple chronic diseases. More details could be found in Table 7.

**Table 6:** Adjusted regression results of health service quality.

	**Depression score**			
	**Model 10**	**Model 11**	**Model 12**	**Model 13**
	**Lower quality**	**Higher quality**	**Lower quality**	**Higher quality**
Chronic disease	1.613^***^	1.395^***^		
	(0.290)	(0.111)		
Multiple chronic diseases			2.284^***^	1.629^***^
			(0.237)	(0.110)
Year (Ref: 2015)	1.029^***^	0.680^***^	1.053^***^	0.721^***^
	(0.228)	(0.094)	(0.228)	(0.094)
Gender (Ref: Female)	−1.626^***^	−1.706^***^	−1.565^***^	−1.676^***^
	(0.347)	(0.158)	(0.343)	(0.158)
Age	−0.022	−0.008	−0.032^**^	−0.013^*^
	(0.016)	(0.007)	(0.016)	(0.007)
**Education (Ref: Primary school and below)**				
Junior high school	−1.875^***^	−0.859^***^	−1.875^***^	−0.846^***^
	(0.300)	(0.136)	(0.297)	(0.136)
High school and above	−1.985^***^	−1.298^***^	−2.020^***^	−1.331^***^
	(0.364)	(0.164)	(0.359)	(0.163)
Marital status (Ref: Others)	−1.422^***^	−1.289^***^	−1.464^***^	−1.283^***^
	(0.392)	(0.179)	(0.389)	(0.179)
Income	−0.113^***^	−0.105^***^	−0.109^***^	−0.106^***^
	(0.029)	(0.013)	(0.029)	(0.013)
Place of residence (Ref: Rural)	−1.153^***^	−1.286^***^	−1.156^***^	−1.313^***^
	(0.287)	(0.127)	(0.284)	(0.127)
**Region (Ref: Eastern region)**				
Central region	0.860^***^	1.300^***^	0.766^***^	1.269^***^
	(0.288)	(0.129)	(0.286)	(0.128)
Western region	0.687^**^	1.827^***^	0.583^*^	1.748^***^
	(0.304)	(0.134)	(0.302)	(0.134)
Sleeping time	−0.697^***^	−0.562^***^	−0.669^***^	−0.557^***^
	(0.058)	(0.025)	(0.057)	(0.025)
Sports (Ref: No)	−0.590^**^	−0.226^**^	−0.568^**^	−0.223^**^
	(0.256)	(0.109)	(0.255)	(0.109)
Smoke (Ref: No)	0.357	0.476^***^	0.339	0.483^***^
	(0.314)	(0.148)	(0.310)	(0.148)
Drink (Ref: No)	−0.602^**^	−0.313^***^	−0.510^**^	−0.303^***^
	(0.258)	(0.112)	(0.256)	(0.112)
Social (Ref: No)	−1.025^***^	−0.465^***^	−1.066^***^	−0.483^***^
	(0.232)	(0.093)	(0.230)	(0.093)
Health insurance (Ref: Not have)	−0.033	−0.223	−0.118	−0.274
	(0.561)	(0.226)	(0.558)	(0.226)
*P*	< 0.001	< 0.001	< 0.001	< 0.001
*R^2^*	0.175	0.166	0.192	0.171
*N*	3,273	15,142	3,273	15,142

Standard errors in parentheses ^*^p < 0.1, ^**^p < 0.05, ^***^p < 0.01. The table showed the moderating effect of health service quality on the relationship between chronic diseases and depression score.

**Table 7:** Adjusted regression results of health service quality.

	**Depression status**			
	**Lower quality**	**Higher quality**	**Lower quality**	**Higher quality**
Chronic disease	0.508^***^	0.386^***^		
	(0.095)	(0.037)		
Multiple chronic diseases			0.558^***^	0.373^***^
			(0.076)	(0.033)
Year (Ref:2015)	0.240^***^	0.203^***^	0.246^***^	0.214^***^
	(0.074)	(0.032)	(0.073)	(0.032)
Gender (Ref: Female)	−0.474^***^	−0.432^***^	−0.460^***^	−0.429^***^
	(0.104)	(0.049)	(0.104)	(0.049)
Age	−0.006	−0.004^**^	−0.007	−0.005^**^
	(0.005)	(0.002)	(0.005)	(0.002)
**Education (Ref: Primary school and below)**				
Junior high school	−0.380^***^	−0.211^***^	−0.381^***^	−0.209^***^
	(0.091)	(0.043)	(0.090)	(0.043)
High school and above	−0.502^***^	−0.313^***^	−0.509^***^	−0.324^***^
	(0.116)	(0.059)	(0.116)	(0.059)
Marital status (Ref: Others)	−0.269^**^	−0.282^***^	−0.277^**^	−0.281^***^
	(0.110)	(0.048)	(0.110)	(0.048)
Income	−0.029^***^	−0.029^***^	−0.029^***^	−0.029^***^
	(0.029)	(0.013)	(0.029)	(0.013)
Place of residence (Ref: Rural)	−0.265^***^	−0.328^***^	−0.267^***^	−0.334^***^
	(0.086)	(0.041)	(0.085)	(0.041)
**Region (Ref: Eastern region)**				
Central region	0.200^**^	0.370^***^	0.181^**^	0.367^***^
	(0.087)	(0.041)	(0.086)	(0.041)
Western region	0.152^*^	0.492^***^	0.133	0.480^***^
	(0.091)	(0.041)	(0.090)	(0.041)
Sleeping time	−0.148^***^	−0.145^***^	−0.140^***^	−0.145^***^
	(0.018)	(0.008)	(0.017)	(0.008)
Sports (Ref: No)	−0.176^**^	−0.079^**^	−0.167^**^	−0.080^**^
	(0.078)	(0.036)	(0.077)	(0.036)
Smoke (Ref: No)	0.099	0.108^**^	0.093	0.110^**^
	(0.094)	(0.046)	(0.094)	(0.046)
Drink (Ref: No)	−0.072	−0.072^**^	−0.053	−0.069^*^
	(0.077)	(0.037)	(0.077)	(0.037)
Social (Ref: No)	−0.167^**^	−0.110^***^	−0.174^**^	−0.112^***^
	(0.069)	(0.030)	(0.069)	(0.030)
Health insurance (Ref: Not have)	−0.018	−0.095	−0.033	−0.106
	(0.151)	(0.068)	(0.150)	(0.068)
*P*	< 0.001	< 0.001	< 0.001	< 0.001
*R^2^*	0.114	0.118	0.124	0.120
*N*	3,273	15,142	3,273	15,142

Standard errors in parentheses ^*^p < 0.1, ^**^p < 0.05, ^***^p < 0.01. The table showed the moderating effect of health service quality on the relationship between chronic diseases and depression status.

## 5. Discussion

This paper aimed to alleviate the effect of chronic disease on depression in middle-aged and elderly in China. Based on the association between chronic disease and depression, this study was the first to analyze the role of health insurance and health service quality through moderating effect in China. Previous studies have analyzed the role of the public health system (26), we added to the empirical evidence suggesting that health insurance and health service quality were favorable factors in alleviating depression in chronic disease patients.

### 5.1. The current situation and trend of depression in middle-aged and elderly people

This study used the panel data from 2011 to 2018 to analyze the temporal trend of depression. It could be seen that the depression score of middle-aged and elderly people in China showed a fluctuating trend from 2011 to 2015, and the increase in the statistical data in 2018 became larger, and the depression score of middle-aged and elderly people in rural area was higher than that in urban area. The depression prevalence increased in 2018 compared to 2011, 2013, and 2015. The potential reasons may be: (1) With the acceleration of the pace of life, the employment and life pressure of middle-aged and elderly people have increased, and their physical functions have deteriorated. Therefore, middle-aged and elderly people were prone to experience psychological stages such as “mid-life crisis” and “menopause,” and were prone to psychological problems. (2) Insufficient attention was paid to the mental health hazards caused by physical health losses such as chronic disease, and mental health care were ignored. (3) In terms of other personal characteristics, since female group may face more family pressures and life chores than male group, they may have higher depression score, and consistent with previous studies (27). Married group had lower depression score than other group, because they were accompanied by their spouses. More educated group was better at regulating their emotions, so their depression score may be lower. Higher income may lead to less stress and lower depression score. The middle-aged and elderly people in rural area had higher depression score than the middle-aged and elderly people in urban area, which was because under the background of China's urban-rural dual structure, rural middle-aged and elderly people had a large gap with urban middle-aged and elderly people in terms of the living environment, convenience and accessibility of public services, which was also consistent with previous study (28). In terms of lifestyle, such as sleep, appropriate alcohol consumption, social interaction and sports could relieve mood and reduce depression score, and smoking would increase depression score.

### 5.2. The association between chronic disease and depression in middle-aged and elderly people

Studies have shown that both chronic disease and multiple chronic diseases could improve depression score and depression status. In the process of chronic disease development, the heterogeneity of individual adjustment was obvious (29). The potential reasons why chronic disease patients were more likely to be depressed may be: (1) Derived from bodily function damage. The potentially harmful coexistence of health-damaging behaviors and loneliness increased the risk of chronic disease (30). For example, chronic disease patients may exhibit a greater fear of activities (31). Patients who suffered from pain frequently were prone to depression and negativity (32). (2) Stemmed from lifestyle disturbance. Chronic pain affected depression through sleeping quality, and pain from chronic disease led to reduce sleeping quality, which in turn led to depression (33). Especially it was in the middle-aged and elderly population that chronic disease increased their depression score by affecting activities of daily living (34). (3) Came from increased economic pressure. In China, the proportion of health expenditure of chronic disease families to the effective family income was 16.3%, that of hypertensive families was 11.4%, and that of normal families was 5.8%, more than 14% of chronic disease families and about 10% of hypertensive families had catastrophic medical expenditures (35). Chronic disease patients had economic pressure due to the long course of disease and the number of visits, which in turn increased the psychological burden and threatened their mental health. The chronic disease could also affect the neglect or abuse by other household members or malnutrition which could also contribute to a higher level of depression (36–38). In general, the existence of chronic disease and multiple chronic diseases would undoubtedly increase the psychological burden of patients from the aspects of body and lifestyle, and may increase the depression score.

### 5.3. Mitigating factors and contributions of depression in middle-aged and elderly patients with chronic disease

Studies have shown that participating in health insurance and higher quality of health service were effective mechanisms for relieving depression from chronic disease in the middle-aged and elderly in China. Comprehensive strategies for chronic disease should not only affect intrinsic motivations such as knowledge and beliefs of patients, but also focused on safeguards such as public health policies.

In terms of health insurance, the reasons for its role may be because: (1) Health insurance was conducive to the transfer of the risk and compensation, and the health insurance amount compensated for economic losses caused by chronic disease. Since 2018, the health insurance poverty alleviation policy has benefited a total of 480 million people in poverty, helped reduce the medical burden by nearly 330 billion yuan, and helped nearly 10 million households who had returned to poverty due to illness out of poverty in China (39). (2) In China, the coverage of health insurance has increased, and the outpatient medical expenses of some chronic diseases with long illness periods and high medical expenses were included in the payment scope of the pooled fund (40), relieving economic and psychological pressure for chronic disease patients.

In terms of health service quality, after entering the new era, people's demands for health protection were increasing. The reasons for the role of health service quality may be: (1) The higher the satisfaction of the middle-aged and elderly patients with the quality of health service, the higher the recognition of the treatment plan, and the more optimistic about the physical function relief, thus helping to relieved depression. (2) The prevailing literature on this subject suggested that most chronic disease patients need help from others in daily life and medical rehabilitation, including emotional support and practical help (29). Helping patients to relieve pain and depressed mood through psychological care in health service were very important, people were regarded as individuals, and person-centered care was considered to be the best way to provide medical care (41). Therefore, the quality of health service was an important aspect of relieving the mood of middle-aged and elderly patients and alleviating the loss of mental health.

### 5.4. Limitation

There were also some limitations in this study: Firstly, we only discussed the differences between having or not having health insurance, and the role of different types of health insurance remains to be discussed. Secondly, the measurement of health service quality in this paper came from the subjective evaluation of patients, which may cause errors due to the patient's condition. Finally, our study concentrated on association, not causation.

## 6. Conclusion

Chronic disease and multiple chronic diseases were important causes of depression in middle-aged and elderly people. Health insurance and the health service quality were the key factors in relieving the depression of chronic disease patients. This paper believed that we should first pay attention to the depression of middle-aged and elderly patients with chronic disease, popularize knowledge of chronic disease, promote disease awareness, and show emotional care for patients with chronic disease. Secondly, we should continue to promote the participation rate of health insurance, and improve the social security system based on endowment insurance and health insurance so as to provide strong medical security for the prevention and treatment of chronic disease in the middle-aged and the elderly. In addition, the quality of health service should be further improved. While improving the level of diagnosis and treatment, the basic and diversified needs of patients with chronic disease should be paid attention to, the accessibility of health service for chronic disease should be improved, and a “patient-centered” health service model should be established.

## Data availability statement

Publicly available datasets were analyzed in this study. This data can be found at: http://charls.pku.edu.cn.

## Ethics statement

The original CHARLS survey data was approved by the Ethical Review Committee of Peking University, and all participants signed informed consent at the time of participation.

## Author contributions

DL: conception and design, data analysis and interpretation, writing-original draft, and supervision. MS: conception and design, funding acquisition, and supervision. XG, BL, and TZ: review and editing. All authors read and approved the final manuscript.
